# High Efficient Photo-Fenton Catalyst of α-Fe_2_O_3_/MoS_2_ Hierarchical Nanoheterostructures: Reutilization for Supercapacitors

**DOI:** 10.1038/srep31591

**Published:** 2016-08-16

**Authors:** Xijia Yang, Haiming Sun, Lishu Zhang, Lijun Zhao, Jianshe Lian, Qing Jiang

**Affiliations:** 1 Key Lab of Automobile Materials, Ministry of Education, College of Materials Science and Engineering, Jilin University, Nanling Campus, Changchun, 130025, P.R. China

## Abstract

A novel three-dimensional (3D) α-Fe_2_O_3_/MoS_2_ hierarchical nanoheterostructure is effectively synthesized via a facile hydrothermal method. The zero-dimensional (0D) Fe_2_O_3_ nanoparticles guide the growth of two-dimensional (2D) MoS_2_ nanosheets and formed 3D flower-like structures, while MoS_2_ facilitates the good dispersion of porous Fe_2_O_3_ with abundant oxygen vacancies. This charming 3D-structure with perfect match of non-equal dimension exhibits high recyclable photo-Fenton catalytic activity for Methyl orange pollutant and nice specific capacity in reusing as supercapacitor after catalysis. The synergistic effect between Fe_2_O_3_ and MoS_2_, the intermediate nanointerfaces, the 3D porous structures, and the abundant oxygen vacancies both contribute to highly active catalysis, nice electrochemical performance and stable cycling. This strategy is simple, cheap, and feasible for maximizing the value of the materials, as well as eliminating the secondary pollution.

The environment and energy issues become more and more prominent, therefore increasing efforts aimed at using renewable energy sources in our daily life has been made^1^,^2^,^3^,^4^,^5^. Semiconductor materials have received intense attentions as a potential solution to the worldwide energy shortage and for environmental purification^6^,^7^,^8^,^9^,^10^,^11^. Among which, hematite (α-Fe_2_O_3_), an intriguing material, is the most stable iron oxide with n-type semiconducting properties under ambient conditions. Owing to the attractive features, such as natural abundance, low cost, nontoxicity, chemical stability, and favorable optical band gap (ca. 2.2 eV), Fe_2_O_3_ has been intensively researched for applications in catalysis^12^,^13^,^14^,^15^, and supercapacitors^16^,^17^. However, it suffers the low efficiency arising from poor absorptive, short hole diffusion length (e.g. 2–4 nm), high electron-hole recombination rate, and poor electrical conductivity^18^,^19^,^20^, which hinder its practical application.

Two-dimensional (2D) layered nanomaterials such as graphene have attracted tremendous research interests in scientific fields because of their unique properties and wide potential applications^21^,^22^,^23^,^24^,^25^. Inspired by the discovery of 2D graphene monolayer and its rich physical phenomenon, MoS_2_, resembling graphene, a typical example of 2D layered nanomaterials, has caused great interest in the past few years^26^,^27^,^28^,^29^,^30^. As a transition metal sulfide, MoS_2_ possesses many excellent properties, and its enhanced visible light absorption, proper band edge, special 2D structures, excellent mechanical and electrical properties make it an ideal candidate to form heterojunctions^31^,^32^. In recent years, several articles have reported the successful synthesis of heterojucions based on MoS_2_ and show unique photocatalytic and electrochemical properties^33^,^34^,^35^,^36^, while few studies can realize the formation of the hybrid of 0D nanoparicles with 2D MoS_2_ nanosheets due to the lack of easy and effective ways to combine them with no agglomeration and high performance.

Based on the above considerations, we design a novel 3D flower-like Fe_2_O_3_/MoS_2_ hierarchical nanoheterostructure firstly through a facile hydrothermal reaction. The Fe_2_O_3_ nanoparticles guide the growth of 2D MoS_2_ to construct the 3D micron-size flowers, while 2D MoS_2_ sheets facilitate the formation and good dispersion of porous Fe_2_O_3_ nanoparticles with abundant oxygen vacancies. The hybrid show excellent photo-Fenton catalytic activity for degrading Methyl orange (MO) which belongs to the azo dyes family and is known to be carcinogenic and mutagenic; meanwhile, exhibit high specific capacity for supercapacitor after 6 successive cycles of photocatalytic reaction. By careful evaluation, we found that the used Fe_2_O_3_/MoS_2_ material show better electrochemical performance after photo-Fenton reaction compared to the fresh materials, which may contribute to the abundant oxygen vacancies formed in the catalytic reaction. This provide an idea for the reapplication of scrap materials to eliminate secondary pollution, decrease the energy consumption and achieve the maximum use of materials, which may eliminate and collectively solve the problems of environment and energy.

## Results

**Figure 1:**
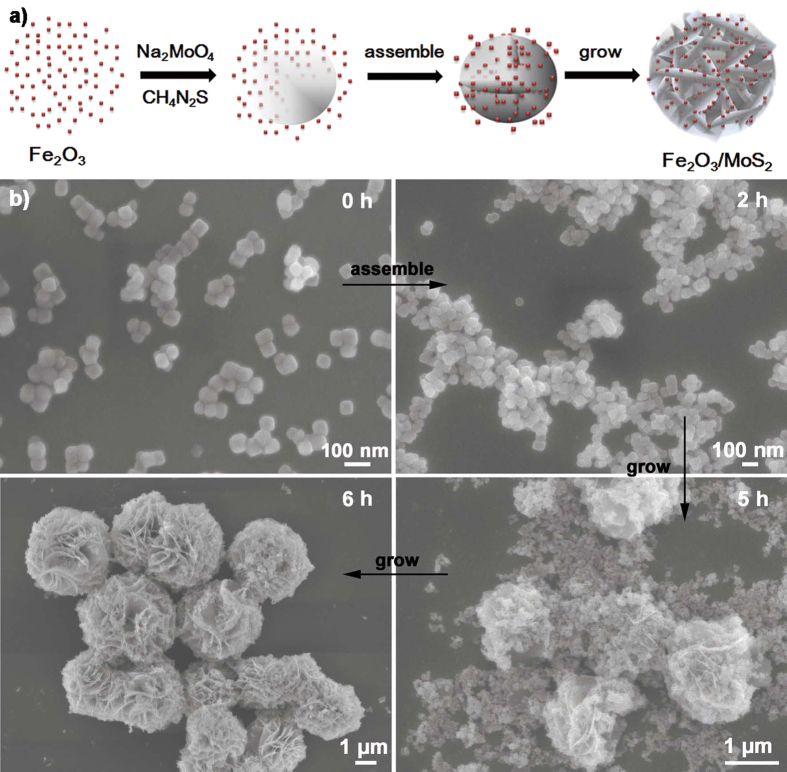
(**a**) Schematic illustration of the 3D α-Fe_2_O_3_/MoS_2_ heterostructures formation; (**b**) morphological characterizations of the formation process of the 3D Fe_2_O_3_/MoS_2_ heterostructures (3.0MF) with different reaction time.

In the reaction, 0D Fe_2_O_3_ nanoparticles were served as the template to assist the growth of 2D MoS_2_ to construct special structure of both Fe_2_O_3_ and MoS_2_. The Fe_2_O_3_ nanoparticles with positive electricity^37^ can gather and absorb the gradually formed negative changed MoS_2_ nanosheets (pH = 7.42)^38^. This facile synthesis strategy for the Fe_2_O_3_/MoS_2_ heterostructures is schematically depicted in Fig. 1. Furthermore, the formed micron-structure with perfect match of non-equal dimension avoids the difficult reclamation compared with nanoscale materials and ensures the cyclic utilization. In the photocatalysis process, H_2_O_2_ was added to the system to develop a photo-Fenton reaction for further improving the catalytic performance. Moreover we design the Fe_2_O_3_/MoS_2_ heterostructures with different weight ratios (MoS_2_: Fe_2_O_3_, 0.6:1.0, 1.0:1.0, 1.4:1.0, 2.0:1.0, 3.0:1.0, 4.0:1.0) and labeled as 0.6MF, 1.0MF, 1.4MF, 2.0MF, 3.0MF and 4.0MF, respectively, to find out the optimal proportion for photocatalysis.

**Figure 2:**
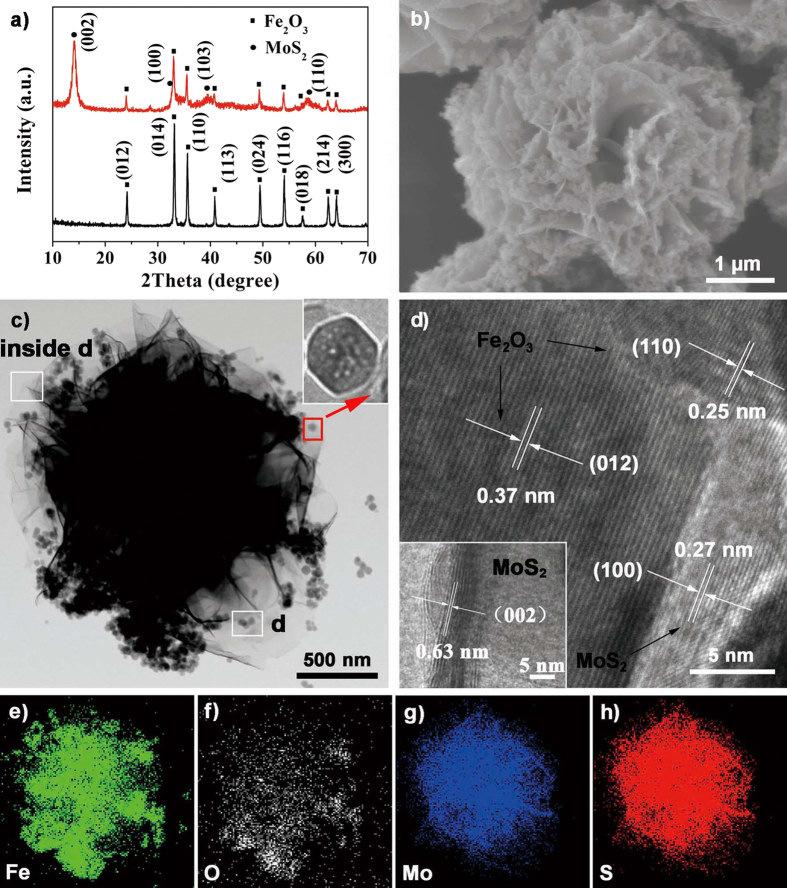
(**a**) XRD patterns of the pre-prepared Fe_2_O_3_ nanoparticles (black curve) and the 3.0MF (red curve); (**b**) SEM, (**c**) TEM, (**d**) HRTEM images of the as prepared 3.0MF; and the corresponding elemental mappings of (**e**) Fe, (**f**) O, (**g**) Mo, and (**h**) S elements; and the inset in (**c**) shows the enlarged view of Fe_2_O_3_ nanoparticles; and the inset in (**d**): HRTEM image of the MoS_2_ sheets.

Figure 2a shows the X-ray diffraction (XRD) patterns of the as-prepared Fe_2_O_3_ nanoparticles and Fe_2_O_3_/MoS_2_ composite, which confirm the formation of MoS_2_ sheet-Fe_2_O_3_ particles hybrid nanostructures. Besides the XRD peaks of Fe_2_O_3_ (JCPDS 33-0664), additional diffraction peaks at 14.1°, 32.7°, 39.5° and 58.3° can be indexed to the (002), (103), (110) and (201) planes of 2H-MoS_2_ (JCPDS 37-1492). Whereas the plane spacing d (002) = 0.63 nm is slightly larger than the standard (0.62 nm) of 2H-MoS_2_, this could be attributed to the lattice distortion of the as-prepared MoS_2_ nanosheets in the presence of Fe_2_O_3_ nanoparticles. The field emission scanning electron microscope (FE-SEM) images shown in Figure S1a reveal that the Fe_2_O_3_ nanoparticles are of cube shape with rounded corner, which have an average edge length of 50 nm. And the 0D Fe_2_O_3_ nanoparticles were uniformly loaded on the 2D MoS_2_ sheets, forming the 3D hierarchical nanoheterostructures with micron-size (Fig. 2b). We can hardly see the Fe_2_O_3_ nanoparticles separating from the MoS_2_ nanosheets, implying a strong interaction between Fe_2_O_3_ and MoS_2_. In the hydrothermal reaction, 2D MoS_2_ sheets support the Fe_2_O_3_ nanoparticles, and facilitate the good dispersion of Fe_2_O_3_, which guarantee the high photocatalytic and electrochemical performance. The morphology varies when alter the proportion of MoS_2_/Fe_2_O_3_, if the proportion is less than 1.4MF, Fe_2_O_3_ nanoparticles can’t be entirely loaded on the MoS_2_ nanosheets. The content of MoS_2_ in the range of 2.0MF~4.0MF ensures the stable 3D-structures with all the Fe_2_O_3_ nanoparticles uniformly loaded on the MoS_2_ flower-like nanosheets, and the flower-like structures with a diameter of ca. 2–4 μm (Figure S2). However, excessive MoS_2_ in the heterostructures might result in lack of the support of Fe_2_O_3_, and make the structures gradually aggregated. When no Fe_2_O_3_ nanoparticles join in, MoS_2_ nanosheets get severe agglomeration (Figure S1b), which could affect the sufficient charge transfer and the efficient photocatalytic and electrochemical activity might be weakened.

To further investigate the detailed structures, Fe_2_O_3_/MoS_2_ heterostructures were characterized by transmission electron microscope (TEM) and high resolution transmission electron microscope (HRTEM). Figure 2c shows the layered structures of MoS_2_ with loaded Fe_2_O_3_ nanoparticles, and the Fe_2_O_3_ nanoparticles formed the porous structures during the hydrothermal reaction with the existence of MoS_2_. The HRTEM image inset Fig. 2d shows the cross section of a MoS_2_ piece (petal) where the layer numbers of MoS_2_ (002) plane is approximately 8–12 with the inter-plane space of 0.63 nm, which has a good match with the XRD analysis. Figure 2d shows the local lattice fringes of a well-defined crystalline Fe_2_O_3_ nanoparticles possessing a lattice spacing of 0.37 and 0.25 nm, which corresponds to the (012) and (103) planes, respectively. The adjacent nanodomain of short-range ordering has a width of ~5 nm with an interlayer spacing of 0.27 nm corresponding to the (100) plane of MoS_2_. Besides, to determine the spatial distributions of the MoS_2_ and Fe_2_O_3_ phase in the heterostructures, the elemental mapping is applied. Figure 2e–h display the existences of Fe, O, Mo, and S elements in the heterostructures, respectively. The O and Fe signals are evenly distributed over the entire heterostructures as expected, certifying that the uniformly loaded Fe_2_O_3_ nanoparticles on the MoS_2_ nanosheets. The EDX analysis (Figure S3) also identifies the existence of the Fe, O, Mo, and S elements. According to the EDX, the molar ratio of Mo: Fe: S: O is 13.46: 27.48: 26.76: 11.68; the results show very low molar ratio of the element O and some difference with the theoretical molar ratio of MoS_2_/Fe_2_O_3_ (MoS_2_/ Fe_2_O_3_ = 1:1).

**Figure 3:**
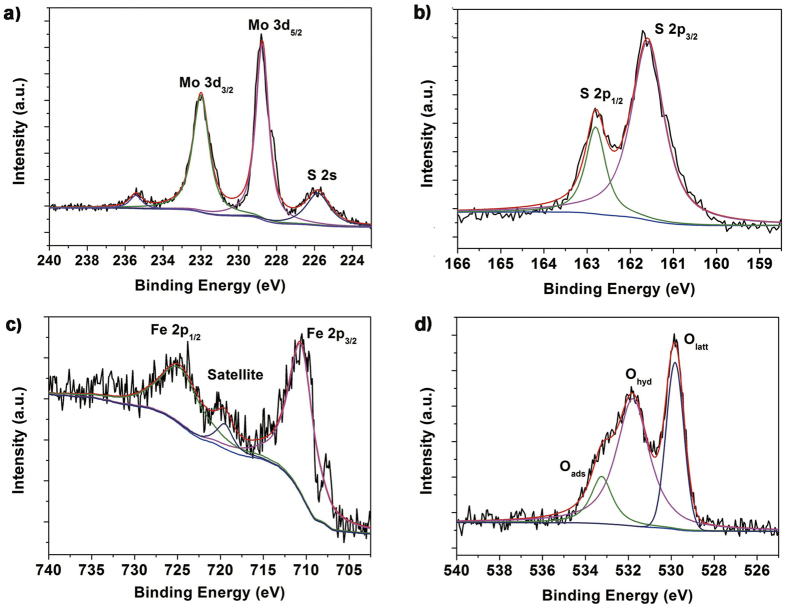
XPS spectra of the 3.0MF: (**a**) Mo 3d and S 2s peaks, (**b**) S 2p peaks, (**c**) Fe 2p peaks, and (**d**) O 1s peaks.

To deeply evaluate the binding behavior of the elements in the sample 3.0MF, XPS spectra for Fe 2p, O 1s, Mo 3d, and S 2p regions were shown in Fig. 3. The binding energies of Mo 3d_3/2_, Mo 3d_5/2_, S 2p_1/2_ and S 2p_3/2_ peaks are located at 232.0, 228.8, 162.8 and 161.6 eV, respectively, suggesting that Mo^4+^ existed in the MoS_2_. The asymmetric peaks and tailing spectra (at about 235.4 eV) for Mo 3d_3/2_ and Mo 3d_5/2_ in the heterostructures are likely due to the existence of a small amount of Mo^6+^. In the high-resolution of Fe 2p spectrum, two distinct peaks at the binding energies of 710.7 eV for Fe 2p_3/2_ and 725.2 eV for Fe 2p_1/2_ with a shake-up satellite at 719.6 eV can be observed, meaning that the iron oxide in the heterostructures should be Fe_2_O_3_, which is in accordance with the result of XRD pattern^39^. The O 1s spectrums could be deconvoluted to three different peaks at the binding energies of 529.8, 531.8, and 533.2 eV, corresponding to the lattice oxygen (O_latt_), hydroxyl oxygen (O_hyd_) and physically adsorbed oxygen (O_ads_), respectively^40^,^41^. It is generally accepted that O_ads_ oxygen species are mainly affected by surface oxygen vacancies since they are often adsorbed only by the oxygen vacancies at vacuum conditions^40^,^42^. Thus, oxygen vacancies should exist on the surface of the couple of MoS_2_ and Fe_2_O_3_, and it’s in conformity to the result of EDX. We supposed that the oxygen vacancies result from the anion exchange reaction occurred in the junction surface: the faster diffusion of O^2−^ than incoming S^2−^ leads to the formation of oxygen vacancies, and then some become to porous structures^43^.

**Figure 4:**
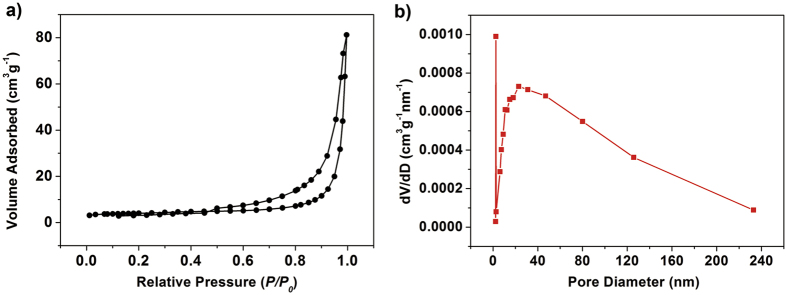
(**a**) N_2_ adsorption–desorption isotherm and (**b**) pore-size distribution curve of the as-obtained 3.0MF.

The nitrogen adsorption-desorption measurement was used to further reveal the porous structures of the as-prepared Fe_2_O_3_/MoS_2_ heterostructures. The shape of hysteresis loops is of type IV, indicating abundant mesopores existing in the heterostructures associated with the overlapping of the 2D MoS_2_ nanosheets. Due to the large size of the heterostructures, the Brunauer–Emmett–Teller (BET) specific surface area of the sample only shows a moderate value of 13.2 m^2^ g^−1^ (Fig. 4a). It seems to prove that the large specific surface area is not the decisive factor of photocatalysis. Besides, the Barrett–Joyner–Halenda (BJH) pore size distribution (Fig. 4b) of the samples indicates the sizes of mesopores have a wide pore-size distribution from 2 to 230 nm. These mesopores work as channels in the photocatalytic process, which assist diffusion of the pollutants in the heterostructures and therefore improve the photocatalytic activity. Meanwhile, the porous Fe_2_O_3_ nanoparticles facilitate electrochemical site and ensures short path lengths of ion diffusion, which enhance the photocatalyst and electrochemical performance.

## Discussion

**Figure 5:**
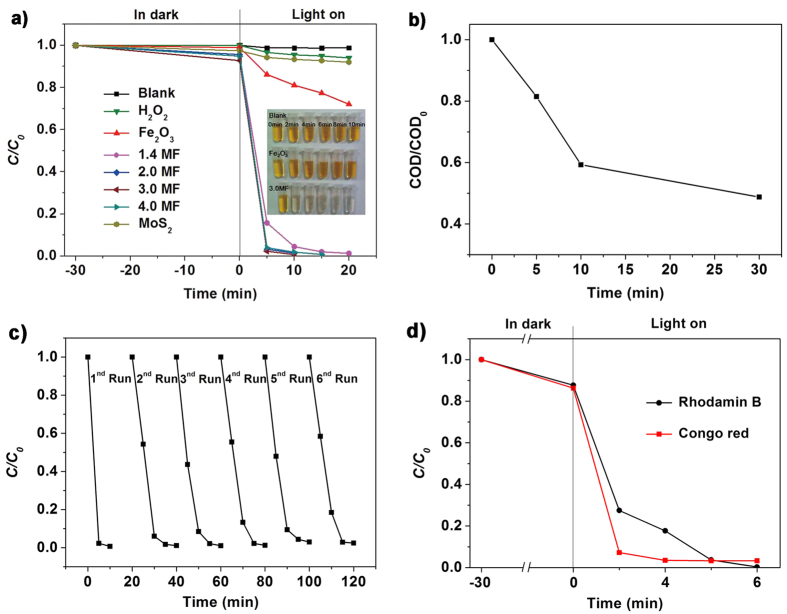
(**a**) Photocatalytic degradation of MO with different catalysts; (**b**) variations in COD during the photocatalytic (3.0MF) degradation of MO in 30 min. The inset (**a**) the photo of the fade of the MO; (**c**) Six cycles of the photocatalytic reduction of MO using sample 3.0MF as the photocatalyst under simulated solar light irradiation for 20 min; (**d**) Photocatalytic degradation of CR (50 mg L^−1^) and RhB (20 mg L^−1^) under simulated solar light with the presence of the 3.0MF.

To demonstrate the photo-Fenton catalytic performance of these unique 3D Fe_2_O_3_/MoS_2_ hierarchical nanoheterostructures, catalytic reduction of MO by the as-obtained Fe_2_O_3_ nanoparticles, MoS_2_ sheets and various Fe_2_O_3_/MoS_2_ heterostructures with H_2_O_2_ under simulated solar light irradiation was investigated. Figure 5a show the change in the concentration of MO (C/C_0_) during the photodegradation process; C_0_ and C are the initial concentration of MO and the measured concentration of MO after photodegradation for a certain time, respectively. For comparison, pure MO and MO with addition of H_2_O_2_ under simulated light irradiation without catalysts were evaluated, but they only show slightly degradation, indicating that the photolysis mechanism of MO and the decomposition capacity of H_2_O_2_ can be ignored. Prior to the light irradiation, the mixed suspension of the catalyst and MO was constant stirred in dark to establish adsorption/desorption equilibrium, and the Fe_2_O_3_/MoS_2_ heterostructures show a slight adsorption of MO. Under simulated solar light irradiation, the sample 3.0MF (75 wt% MoS_2_) shows the highest photocatalytic activity with 99% of MO degraded within 10 min, which is one of the most effective photocatalyst to our knowledge^15^,^36^,^44^,^45^,^46^. The other Fe_2_O_3_/MoS_2_ heterostructures also show excellent photocatalytic activity, which is significantly higher than those of pure Fe_2_O_3_ and MoS_2_.

To quantitatively estimate the reaction kinetics of the MO degradation, the degradation rate is calculated based on the Fig. 5a, and the constants k of Fe_2_O_3_, MoS_2_, 1.4MF, 2.0MF, 3.0MF, and 4.0MF are 0.01485, 0.00262, 0.175, 0.18021, 0.2301, and 0.16411 per min, respectively (Figure S4)^47^,^48^. One can see that all the Fe_2_O_3_/MoS_2_ heterostructures exhibit much higher photocatalytic activities than the pure Fe_2_O_3_ and MoS_2_. And the 3.0MF found to exhibits the highest rate, about 87 times higher than that of MoS_2_ and 15 times higher than that of Fe_2_O_3_. Therefore, the combination of Fe_2_O_3_ and MoS_2_ with nanoheterostructures makes an effective way to significantly enhance the photocatalytic activity. Figure 5b shows the normalized Chemical Oxygen Demand (COD) removal during the photocatalytic treatment with the 10 mg catalysts. It is observed that more than 56% COD of wastewater can be reduced only after 30 min of exposure to the simulated solar light in the presence of the 3.0MF. These results point out the fast mineralization rate of Fe_2_O_3_/MoS_2_ heterostructures under simulated solar light radiation.

The decent photocatalytic performance could be explained as follows. One is the relatively high adsorption capacity within the range of visible and ultraviolet light. Meanwhile the as prepared architecture with channels has certain of absorption property can contact with the contaminants more sufficiently. Besides, when Fe_2_O_3_ nanoparticles loaded on the surface of the MoS_2_ nanosheets, oxygen vacancies, efficient interfaces between Fe_2_O_3_ and MoS_2_ as well as the heterostructures were developed, which benefit the process of electron-hole separation. Also the UV-vis absorption spectra (Figure S5) affirm the good photocatalytic performance of the heterostructures. For Fe_2_O_3_ nanoparticles, the effective light absorption extends from UV to visible region (around 500 nm), while MoS_2_ nanosheets show significantly increased absorption in visible light wavelength region larger than 500 nm. Therefore, the combination of Fe_2_O_3_/MoS_2_ presents a considerable absorption in whole UV-vis wavelength range, and increases the photocatalytic efficiency.

Based on Figure S5, the conduction band edge (E_CB_) and the valence band edge (E_VB_) of the Fe_2_O_3_ and MoS_2_ at the point of zero charge can be estimated by the Equations (1) and (2) according to the electronegativity^49^:


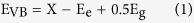



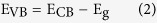


where X is the semiconductor’s absolute electronegativity, and the values of X for Fe_2_O_3_ and MoS_2_ are 5.88 and 5.32 eV, respectively; E_e_ is the energy of free electrons on the hydrogen scale (ca. 4.5 eV); E_VB_ is the valence band (VB) edge potential; E_CB_ is the conduction band (CB) edge potential and E_g_ is the band gap of the semiconductor. By extrapolating the straight portion of the (Ahν)^2^−hν plot to the x axis, we get the values of band gaps through the intersection, which are 1.97 and 1.93 eV for Fe_2_O_3_ and MoS_2_, respectively (Figure S6). Therefore, the calculated CB and VB edge positions are 2.37 and 0.39 eV for Fe_2_O_3_, and 1.79 and −0.14 eV for MoS_2_, respectively (Figure S7a).

The potentials of CB and VB of MoS_2_ are more negative than those of Fe_2_O_3_, the staggered alignment of band edges at the heterointerface can improve spatial charge separation of the photogenerated electron and hole in different parts of the heterostructures. Under simulated solar light irradiation, the photogenerated electrons in the CB of MoS_2_ transfer to the CB of Fe_2_O_3_, for the other band, the leaving holes will transfer from the VB of Fe_2_O_3_ to the VB of MoS_2_ in opposite direction. The electrons and holes transfer spontaneously in the heterostructures, therefore the yield and lifetime of the photo-induced electron/hole increased while reducing the chance for their recombination, hence the photocatalytic performance get improved.

In order to investigate the separation efficiency of photogenerated electrons and holes in our system, the electrochemical impedance spectroscopy (EIS) was applied. Figure S7b shows the EIS Nyquist plots of Fe_2_O_3_, MoS_2_ and Fe_2_O_3_/MoS_2_ photocatalysts with and without irradiation. The radius of the arc on the EIS spectra reflects the reaction rate occurring at the surface of the electrode^50^, and the EIS Nyquist plot with smaller arc radius indicates the faster interfacial charge transfer and more effective separation of photogenerated electron–hole pair. The arc radius on the EIS Nyquist plot of MoS_2_ is small under light irradiation, suggesting the faster interfacial charge transfer on the MoS_2_ sheets, meanwhile arc radius of the composite of Fe_2_O_3_/MoS_2_ is smaller than that of Fe_2_O_3_ with and without irradiation, suggesting that the combination of Fe_2_O_3_ and MoS_2_ made the easier charge transfer. This result indicated that the Fe_2_O_3_/MoS_2_ heterostructures can effectively enhance the separation efficiency of photogenerated electron–hole pairs, and therefore enhance the photocatalytic activity.

In our Fe_2_O_3_/MoS_2_ heterostructures system, H_2_O_2_ is added to further enhance the contaminations degradation. The photo-Fenton catalytic process formed, and the reactions between iron ion and H_2_O_2_ are proceeding at the same time. Irradiation with sunlight, Fe^3+^ would be reduced to Fe^2+^ and generate the •OH in water surroundings^51^,^52^. Followed by, Fe^2+^ would react with the adsorbed oxygen molecules on the surface of the heterostructures to form oxidizing species (O_2_•−). The superoxide anion radicals (O_2_•−) which on protonation generate the hydroperoxy (HO_2_•) radicals and subsequently produce hydroxyl radicals •OH. Meanwhile, in the presence of H_2_O_2_, Fe^2+^ is easily oxidized to Fe^3+^ and the •OH generates at the same time. So the regenerated Fe^3+^/Fe^2+^ cycles formed, which make more strong oxidant •OH for the photocatalytic degradation.

**Figure 6:**
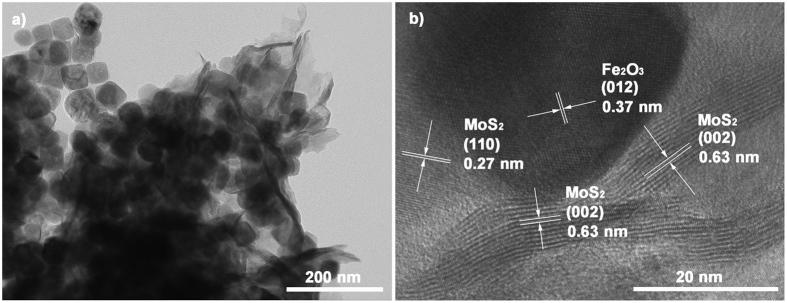
(**a**) TEM and (**b**) HRTEM images of the recycled 3.0MF.

We have further studied the stability and reusability of the as prepared heterostructures (3.0MF) by collecting and reusing the same photocatalyst for 6 cycles (Fig. 5c). Although incomplete collection of the photocatalyst during each step, the 3.0MF retains 97% degradation degree of MO with 20 min of solar light irradiation even after 6 successive cycles, indicating its high stability and great promise in future practical applications. The TEM and HRTEM images shown in the Fig. 6 reveal the phase of the cycled catalyst has not changed, and the structures basically maintained intact as compared with Figure S8, while part of the MoS_2_ nanosheets suffer the photo corrosion, which can be further confirmed by the XRD pattern and XPS spectra (Figure S9 and Figure S10). Even part of the MoS_2_ suffers the photo corrosion; the cycled catalyst still maintained high stability photocatalytic activity. We propose a reaction mechanism that at the process of hydrothermal treatment, anion exchange reaction occurred; small amount of S^2−^ replaced the O^2−^ in Fe_2_O_3_. When irradiated by the solar light, some Fe-S were photo corroded, subsequent vacancies formed, which act as the catchers for electrons. Also, the ratio of O_ods_/O_latt_ is evident as shown in Figure S10d, confirming the oxygen vacancies formed in the photo-Fenton catalytic process. Hence, separation of the electrons and holes is promoted, which could cause the high reusability of the photocatalyst. But the problem of photo corrosion for MoS_2_ still needs to be solved and improved.

Besides, the generalizability of the Fe_2_O_3_/MoS_2_ heterostructures was also evaluated by the degradation of Congo red (CR, 50 mg L^−1^) and Rhodamine B (RhB, 20 mg L^−1^) (Fig. 5d). CR, an anionic azo dye with one central biphenyl group and two symmetric naphtalenic groups are difficult to biodegrade due to their complex aromatic structures; RhB, a cation dye, which can well dissolve in water or organic solvent and has been found to be potentially toxic and carcinogenic and show good stability. However, it is noticeable that the CR and the RhB were degraded within 4 min and 6 min, respectively by introduce the catalyst. Overall, compared with those of previously reported excellent photocatalysts (Table S1), the Fe_2_O_3_/MoS_2_ heterostructures are shows high-level photocatalytic abilities, so the Fe_2_O_3_/MoS_2_ heterostructures have high potential in practical applications.

**Figure 7:**
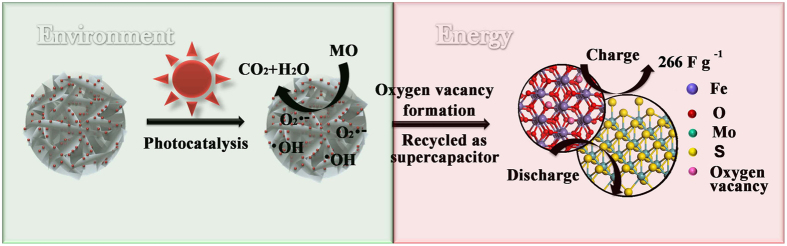
Schematic diagram of the catalyst recycled as the electrode for supercapacitors.

**Figure 8:**
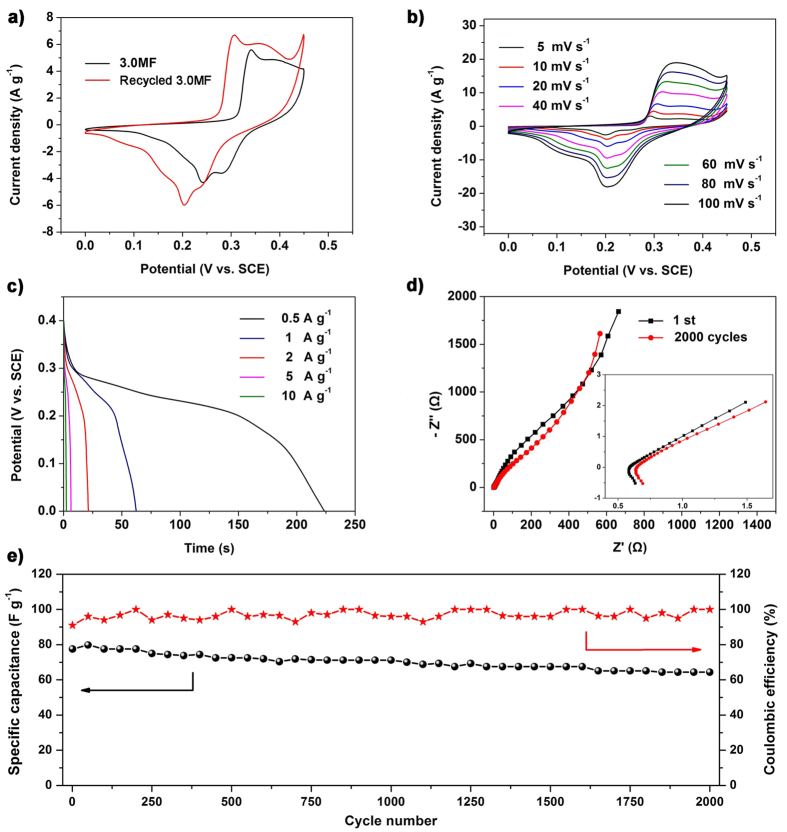
(**a**) CV curves of the 3.0MF and recycled 3.0MF at a scan rates 20 mv s^−1^; (**b**) CV curves of the recycled 3.0MF at different scan rates; (**c**) discharge curves of the recycled 3.0MF at different current densities; (**d**) EIS spectra of the recycled 3.0MF before and after the durability testing; (**e**) cycle performance of the recycled 3.0MF at 5 A g^−1^ for 2000 cycles.

As we know, few researches concerned with the reapplication of the abandoned catalysts to eliminate the secondary pollution and decrease the energy consumption. In this work, we collected the materials after 6 cycles’ catalysis and applied as the electrode for supercapacitors to reach the maximize value recapture of the materials (Fig. 7). The electrochemical performance of the recycled catalyst was tested in a three-electrode system and shown in Fig. 8. The cyclic voltammetry (CV) curves of the 3.0MF and recycled 3.0MF electrodes at a scan rate of 20 mV s^−1^ are shown in Fig. 8a. Obviously, the recycled 3.0MF shows higher current density and larger CV curve area than those of the 3.0MF, suggesting that the photocatalytic process activates the 3.0MF and improves the electrochemical performance. Figure 8b shows the CV curves of recycled 3.0MF, recorded in the potential window from 0 to 0.45 V at different scan rates. A pair of remarkable redox peaks can be observed in each curve, indicating that the capacitive characteristics are mainly influenced by the faradic reduction/oxidation reaction. With the increase of the scan rate, the intensities of the redox peaks got strengthened, and the anodic peak shifted towards the positive potential, while the cathodic peak shifted towards the negative potential. Remarkably, the peak potential shifts only ca. 53 mV for a 20-time increase in the scan rate, indicating the low polarization for our electrode. What’s more, even at a high scan rate of 100 mV s^−1^, the curve still shows the evident redox peaks and the shape basically remains unchanged, suggesting its potential as the electrochemical supercapacitors and its nice electrical conductivity even after 6 cycles’ photocatalysis.

Figure 8c shows the galvanostatic discharging curves of the recycled 3.0MF at different current densities. From the discharge curves at a discharge density of 0.5 A g^−1^ and 1 A g^−1^, the specific capacitances of the recycled 3.0MF were calculated to be 266 F g^−1^ and 150 F g^−1^, respectively. Furthermore, the recycled 3.0MF showed nice rate performance with a capacitance of 62 F g^−1^ retained at a current density as high as 10 A g^−1^. The nice specific capacitance can be mainly attributed to the porous structures of the Fe_2_O_3_ nanoparticles, which facilitate electrochemical sites; short ion diffusion path lengths and more paths for insertion and extraction ions. Meanwhile, the Fe_2_O_3_ owns rich oxygen vacancies enable remarkably improved conductivity, increased active sites and highly reversible, faster charge transfer kinetics^16^,^53^. Furthermore, even part of the MoS_2_ suffers the photo corrosion, synergistic effect between MoS_2_ and Fe_2_O_3_ still promote the materials’ electrochemical performance. Compared with the recycled 3.0MF (150 F g^−1^ at 1 A g^−1^), the specific capacitance of the 3.0MF is 94.75 F g^−1^ at 1 A g^−1^, further confirming the vital function of oxygen vacancies in the electrochemical process (Figure S11a). Considering the influence of the loss of MoS_2_, we tested the sample 2.0MF and 1.4MF for electrochemical performance (Figure S12) and their capacitance are 96 F g^−1^ and 104 F g^−1^ at 1 A g^−1^, respectively, The results did not show much enhancement compared with the 3.0MF, and the performance is lower than the recycled 3.0MF. Therefore, the oxygen vacancies are supposed to be formed in the photo-Fenton catalytic process, and play important role in the electrochemical performance improvement.

The EIS of the recycled 3.0MF before and after the durability testing and 3.0MF were carried out within the frequency range of 100 kHz to 0.01 kHz and shown in the Fig. 8d and Figure S11b in terms of the Nyquist plot. The recycled 3.0MF after photocatalytic process show lower equivalent series resistance (ESR) of 0.59 Ω compared with the 0.67 Ω for 3.0MF, again shows the improved conductivity of the recycled 3.0MF after introduction of oxygen vacancies. Besides, the plot in Fig. 8d shows no significant difference for the recycled 3.0MF before and after 2000 cycles, and it owns low equivalent series resistance (ESR) at 0.64 Ω even after 2000 cycles, indicating its excellent stability property. It is suggesting the high electronic conductivity and fast charge transfer of the recycled 3.0MF, further indicating its potential for reapplication as the electrode of the supercapacitors.

We also examined the durability of the recycled 3.0MF electrode. The charge/discharge cycle life was measured to evaluate the cycle performance of the electrode. As shown in Fig. 8e, the specific capacitance of the electrode presents a slight increase in the first 50 cycles probably due to the activation process, and then decreases gradually thereafter with 82% retention after 2000 cycles at 5 A g^−1^, indicating its good cycling stability.

The technique recycled the used photocatalystas electrode material for supercapacitor has been proved reasonable and feasible through verification test and compared with other work^54^,^55^,^56^,^57^,^58^,^59^. The recycled photocatalyst shows much better electrochemical performance than that of fresh material due to the presence of rich oxygen vacancies on the surface of used photocatalyst during the photocatalytic process. The way to recycle the waste materials as energy sources is feasible, and may be an effective and a promising method to solve the serious problems of environment and energy at the same time.

## Conclusion

In conclusion, we have first demonstrated the construction of Fe_2_O_3_/MoS_2_ heterostructures with perfect match of non-equal dimension which show high-performance as the photocatalyst for degradation of several organic dyes. Due to the oxygen vacancies formed in the photo-Fenton catalytic process, the material after catalyst shows better specific capacitance of 266 F g^−1^ compared with the fresh materials, meanwhile possesses good stability with 84% of the initial capacitance remaining after 2000 cycles. Therefore, our findings have therefore opened up a new look for improving the photocatalytic activity, meanwhile put forward a green and feasible way for photocatalyst reapplication, which may build a bridge for environment and energy in the near future.

## Methods

### Synthesis of the Fe_2_O_3_/MoS_2_ heterostructures

All chemicals of analytical grade purity were used as starting materials without further purification.

The Fe_2_O_3_ nanocube with rounded corner was prepared by modifying a method developed by Fang *et al*.^36^. In a typical experiment, ferric chloride hexahydrate (0.676 g) was dissolved in ethanol (10 mL) under sonication, then deionized water (1.7 mL) added to the above solution under vigorous stirring, followed by sodium acetate (2 g) was added to the mixture solution. After stirring for 1 h, the reaction mixture was sealed in a 50 mL of Teflon-lined stainless steel autoclave, kept at 200 °C for 24 h and then cooled to room temperature. The resulting Fe_2_O_3_ nanocube was collected by centrifugation, then washed with deionzed water and ethanol several times and dried in a vacuum oven at 60 °C for 12 h.

The flower-like Fe_2_O_3_/MoS_2_ heterostructures were synthesized by a facile hydrothermal method. Na_2_MoO_4_·2H_2_O (180 mg) and CH_4_N_2_S (360 mg) were dissolved in deionized water (40 mL), and then the prepared Fe_2_O_3_ nanocube (40 mg) was added into the solution. After sonication for 30 min, the homogeneous solution was transferred into a 50 mL of Teflon-lined autoclave and kept at 200 °C for 24 h. The resulting Fe_2_O_3_/MoS_2_ heterostructures were collected by centrifugation, then washed with deionized water and ethanol several times and dried in a vacuum oven at 60 °C for 12 h. Similarly, a series of the Fe_2_O_3_/MoS_2_ heterostructures were prepared through adjusting the mass ratio by varying the amount of MoS_2_. The pure MoS_2_ planes were prepared by the same procedure without the introduction of Fe_2_O_3_ for comparison.

### Characterizations

The phases of the α-Fe_2_O_3_/MoS_2_ heterostructures were analyzed by Powder X-ray diffraction (XRD, Rigaku D/MAX 2500PC), and the XRD patterns were collected from 10° to 70° in 2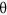 with a Cu target and a mono-chronometer at 40 kV and 250 mA. A field emission scanning electron microscope (FE-SEM, JSM−6700F) was used to characterize the morphologies and size of the synthesized samples. A tungsten lamp was employed and the acceleration voltage was 10 kV. Transmission electron microscope (TEM, JEM-2100F) and corresponding energy dispersive X-ray (EDX) spectrometry were applied for the detailed microstructure and composition analyses, and the amorphous carbon coated copper grids were used as the sample supporters. X-ray photoelectric spectrum (XPS) with an ESCALAB Mk II (Vacuum generators) spectrometer using Al Kα X-ray (240 W) was applied to detect the electronic states of elements in the samples. N_2_ adsorption/desorption isotherms were used to determine the surface areas and porosity of the sample on a Micromeritics ASAP 2020M, and all the samples has been degassed at 110 °C for 12 h in vacuum before the test. The photoelectrochemical measurements were measured on an electrochemical system (CHI-660B, China), using a conventional three-electrode cell. Saturated calomel electrode (SCE) works as the reference electrode, a Pt sheet as the counter electrode (area, 2.0 × 2.0 cm^2^), 3.0MF/ITO (indium-oxide) and Fe_2_O_3_/ITO electrode as the working electrodes. To prepare 3.0MF/ITO and Fe_2_O_3_/ITO electrode, ITO plate was coated with the 3.0MF and Fe_2_O_3_ slurry respectively, which containing solid PVDF and N-methyl-2-pyrrolidone (NMP) solvent. And the weighted ratio in the solid was 9:1 (3.0MF or Fe_2_O_3_: PVDF). Finally, the working electrodes dried at 60 °C for 6 h to evaporate the excess NMP, yielding the working electrode.

### Photocatalysis Test

The photocatalytic activities of the as-prepared Fe_2_O_3_/MoS_2_ were evaluated by the degradation of 20 mg L^−1^ MO solution under simulated solar light irradiation derived from a 300 W Xenon lamp (CEL-HXUV300) with an AM 1.5 filters in air at ambient temperature. Typically, 10 mg of the catalysts were added to the 30 mL MO (20 mg L^−1^) solution, and prior to the light irradiation, the suspension of photocatalyst and MO was stirred for 30 min in dark to establish the adsorption/desorption equilibrium. Then 0.4 mL H_2_O_2_ was added to the solution, and the mixture was exposed to the simulated solar light irradiation with the constant stirring. At given time intervals of illumination, the reaction mixture was centrifuged, and measured on the ultraviolet–visible (UV-vis) spectrophotometer (UV-6100PC). Furthermore, the absorption spectra of the samples were measured on a UV–vis spectrophotometer (TU-1901) using BaSO_4_ as a reference.

### Electrochemical Tests

The working electrodes for cyclic voltammetry (CV), charge-discharge and electrochemical impedance spectroscopy (EIS) were prepared by mixing the recycled 3.0MF with carbon black and polyvinylidene difluoride (PVDF) at a weight ratio of 8:1.5:0.5. After thorough mixing by stirring for 6 h, the slurry is pasted onto a piece of nickel foam and then dried at 110 °C in a vacuum oven overnight. The dried nickel foams were pressed to be a thin foil at a pressure of 10 MPa for 30 s, and the loading mass of the active materials on Ni foam current collector was around 2 mg cm^−2^. The electrochemical measurements were measured on an electrochemical system (CHI-660B, China), using a conventional three-electrode cell in 3.0 M KOH solution. Saturated calomel electrode (SCE) works as the reference electrode, a Pt sheet as the counter electrode (area, 2.0 × 2.0 cm^2^).

## Additional Information

**How to cite this article**: Yang, X. *et al*. High Efficient Photo-Fenton Catalyst of α-Fe_2_O_3_/MoS_2_ Hierarchical Nanoheterostructures: Reutilization for Supercapacitors. *Sci. Rep.* **6**, 31591; doi: 10.1038/srep31591 (2016).

## Supplementary Material

Supplementary Information

## References

[b1] Wentuan Bi. . Molecular co-catalyst accelerating hole transfer for enhanced photocatalytic H_2_ evolution. Nat. commun. 6, 1–7 (2015). 10.1038/ncomms9647 PMC4639900 26486863

[b2] Cao X. . Metal oxide-coated three-dimensional graphene prepared by the use of metal–organic frameworks as precursors. Angew. Chem. Int. Ed. 53, 1404–1409 (2014). 10.1002/anie.201308013 24459058

[b3] Yu J. . Metallic fabrics as the current collector for high-performance graphene-based flexible solid-State supercapacitor. ACS appl. Mater. interfaces 8, 4724–4729 (2016). 26830192 10.1021/acsami.5b12180

[b4] Li H. . Electrochemical doping of anatase TiO_2_ in organic electrolytes for high-performance supercapacitors and photocatalysts. J. Mater. Chem. A 2, 229–236 (2014).

[b5] Huang Z. F. . Tungsten oxides for photocatalysis, electrochemistry, and phototherapy. Adv. Mater. 27, 5309–5327 (2015). 26287959 10.1002/adma.201501217

[b6] Li L. . A general strategy toward carbon cloth-based hierarchical films constructed by porous nanosheets for superior photocatalytic activity. Small 11, 2429–2436 (2015). 25604389 10.1002/smll.201403582

[b7] Lu Y. . Facile synthesis of graphene-like copper oxide nanofilms with enhanced electrochemical and photocatalytic properties in energy and environmental applications. Acs Appl. Mater. Interfaces. 7, 9682–9690 (2015). 25901466 10.1021/acsami.5b01451

[b8] Liao Q., Li N., Jin S., Yang G. & Wang C. All-solid-state symmetric supercapacitor based on Co_3_O_4_ nanoparticles on vertically aligned graphene. ACS Nano 9, 5310–5317 (2015). 25938705 10.1021/acsnano.5b00821

[b9] Liu L., Sun W., Yang W., Li Q. & Shang J. K. Post-illumination activity of SnO_2_ nanoparticle-decorated Cu_2_O nanocubes by H_2_O_2_ production in dark from photocatalytic “memory”. Sci. rep. 6, 1–11 (2016). 10.1038/srep20878 PMC4754728 26879006

[b10] Wang C. C., Hsueh Y. C., Su C. Y., Kei C. C. & Perng T. P. Deposition of uniform Pt nanoparticles with controllable size on TiO_2_-based nanowires by Atomic layer deposition and their photocatalytic properties. Nanotechnology 26, 254002 (2015). 26041474 10.1088/0957-4484/26/25/254002

[b11] Xu D., Cheng B., Cao S. & Yu J. Enhanced photocatalytic activity and stability of Z-scheme Ag_2_CrO_4_-GO composite photocatalysts for organic pollutant degradation. Appl. Catal. B 164, 380–388 (2015).

[b12] Mou X. . Rod-shaped Fe_2_O_3_ as an efficient catalyst for the selective reduction of nitrogen oxide by ammonia. Angew. Chem. Int. Ed. 51, 2989–2993 (2012). 10.1002/anie.201107113 22311597

[b13] Shi F. . Tuning catalytic activity between homogeneous and heterogeneous catalysis: improved activity and selectivity of free nano-Fe_2_O_3_ in selective oxidations. Angew. Chem. Int. Ed. 46, 8866–8868 (2007). 10.1002/anie.200703418 17924600

[b14] Han J. . Investigation of the facet-dependent catalytic performance of Fe_2_O_3_/CeO_2_ for the selective catalytic reduction of NO with NH_3_. J. Phys. Chem. C 120, 1523–1533 (2016).

[b15] Yun S., Lee Y. C. & Park H. S. Phase-controlled iron oxide nanobox deposited on hierarchically structured graphene networks for lithium ion storage and photocatalysis. Sci. rep. 6, 1–9 (2016). 10.1038/srep19959 PMC4731794 26821937

[b16] Lu X. . Oxygen-deficient hematite nanorods as high-performance and novel negative electrodes for flexible asymmetric supercapacitors. Adv. Mater. 26, 3148–3155 (2014). 24496961 10.1002/adma.201305851

[b17] Gund G. S. . Low-cost flexible supercapacitors with high-energy density based on nanostructured MnO_2_ and Fe_2_O_3_ thin films directly fabricated onto stainless steel. Sci. rep. 5, 1–13 (2015). 10.1038/srep12454 PMC4513645 26208144

[b18] Zhang J. Z. Interfacial charge carrier dynamics of colloidal semiconductor nanoparticles. J. Phs. Chem. B 104, 7239–7253 (2000).

[b19] Jorand Sartoretti C. . Photoelectrochemical oxidation of water at transparent ferric oxide film electrodes. J. Phs. Chem. B 109, 13685–13692 (2005). 10.1021/jp051546g 16852715

[b20] Zhang M. . Improving hematite’s solar water splitting efficiency by incorporating rare-earth upconversion nanomaterials. J. Phys. Chem. Lett. 3, 3188–3192 (2012). 26296027 10.1021/jz301444a

[b21] Xiao F. X., Miao J. & Liu B. Layer-by-layer self-assembly of CdS quantum dots/graphene nanosheets hybrid films for photoelectrochemical and photocatalytic applications. J. Am. Chem. Soc. 136, 1559–1569 (2014). 24392972 10.1021/ja411651e

[b22] Xiang Q., Yu J. & Jaroniec M. Synergetic effect of MoS_2_ and graphene as cocatalysts for enhanced photocatalytic H_2_ production activity of TiO_2_ nanoparticles. J. Am. Chem. Soc. 134, 6575–6578 (2012). 22458309 10.1021/ja302846n

[b23] Morin S. A., Forticaux A., Bierman M. J. & Jin S. Screw dislocation-driven growth of two-dimensional nanoplates. Nano Lett. 11, 4449–44455 (2011). 21894947 10.1021/nl202689m

[b24] Stankovich S. . Graphene-based composite materials. Nature 442, 282–286 (2006). 16855586 10.1038/nature04969

[b25] Huang X., Zeng Z., Fan Z., Liu J. & Zhang H. Graphene-based electrodes. Adv. Mater. 24, 5979–6004 (2012). 22927209 10.1002/adma.201201587

[b26] Radisavljevic B., Radenovic A., Brivio J., Giacometti V. & Kis A. Single-layer MoS_2_ transistors. Nature Nanotech. 6, 147–150 (2011). 10.1038/nnano.2010.279 21278752

[b27] Splendiani A. . Emerging photoluminescence in monolayer MoS_2_. Nano Lett. 10, 1271–12275 (2010). 20229981 10.1021/nl903868w

[b28] Li H., Wu J., Yin Z. & Zhang H. Preparation and applications of mechanically exfoliated single-layer and multilayer MoS_2_ and WSe_2_ nanosheets. Acc. Chem. Res. 47, 1067–1075 (2014). 24697842 10.1021/ar4002312

[b29] Zhang W. . Ultrahigh-gain photodetectors based on atomically thin graphene-MoS_2_ heterostructures. Sci. Rep. 4, 1–8 (2014). 10.1038/srep03826 PMC3899643 24451916

[b30] Zhu C., Mu X., van Aken P. A., Yu Y. & Maier J. Single-layered ultrasmall nanoplates of MoS_2_ embedded in carbon nanofibers with excellent electrochemical performance for lithium and sodium storage. Angew. Chem. Int. Ed. 53, 2152–2156 (2014). 10.1002/anie.201308354 24446245

[b31] Zhou W. . Synthesis of few-layer MoS_2_ nanosheet-coated TiO_2_ nanobelt heterostructures for enhanced photocatalytic activities. Small 9, 140–147 (2013). 23034984 10.1002/smll.201201161

[b32] Liu Y., Yu Y. X. & Zhang W. D. MoS_2_/CdS heterojunction with high photoelectrochemical activity for H_2_ evolution under visible light: the role of MoS_2_. J. Phys. Chem. C 117, 12949–12957 (2013).

[b33] Hu L., Ren Y., Yang H. & Xu Q. Fabrication of 3D hierarchical MoS_2_/polyaniline and MoS_2_/C architectures for lithium-ion battery applications. Acs Appl. Mater. Interfaces. 6, 14644–14652 (2014). 25100439 10.1021/am503995s

[b34] Zhao H. . *In situ* light-assisted preparation of MoS_2_ on graphitic C_3_N_4_ nanosheets for enhanced photocatalytic H_2_ production from water. J. Mater. Chem. A 3, 7375–7381 (2015).

[b35] Xie B. Q. . Hydrothermal synthesis of layered molybdenum sulfide/N-doped graphene hybrid with enhanced supercapacitor performance. Carbon 99, 35–42 (2016).

[b36] Han S. . One-step hydrothermal synthesis of 2D hexagonal nanoplates of α-Fe_2_O_3_/graphene composites with enhanced photocatalytic activity. Adv. Funct. Mater. 24, 5719–5727 (2014).

[b37] Breeuwsma A. & Lyklema J. Interfacial electrochemistry of haematite (α-Fe_2_O_3_). Discuss. Faraday Soc. 52, 324–333 (1971).

[b38] Heising J. & Kanatzidis M. G. Exfoliated and restacked MoS_2_ and WS_2_: ionic or neutral species? encapsulation and ordering of hard electropositive cations. J. Am. Chem. Soc. 121, 11720–11732 (1999).

[b39] Grosvenor A. P., Kobe B. A., Biesinger M. C. & McIntyre N. S. Investigation of multiplet splitting of Fe 2p XPS spectra and bonding in iron compounds. Surf. Interface Anal. 36, 1564–1574 (2004).

[b40] Liu Y. . Controlled preparation and high catalytic performance of three-dimensionally ordered macroporous LaMnO_3_ with nanovoid skeletons for the combustion of toluene. J. Catal. 287, 149–160 (2012).

[b41] Machocki A. . Manganese–lanthanum oxides modified with silver for the catalytic combustion of methane. J. Catal. 227, 282–296 (2004).

[b42] Barbero B. P., Gamboa J. A. & Cadús L. E. Synthesis and characterisation of La_1−x_Ca_x_FeO_3_ perovskite-pype oxide catalysts for total oxidation of volatile organic compounds. Appl. Catal. B 65, 21–30 (2006).

[b43] Park J., Zheng H., Jun Y. W. & Alivisatos A. P. Hetero-epitaxial anion exchange yields single-crystalline hollow nanoparticles. J. Am. Chem. Soc. 131, 13943–13945 (2009). 19788329 10.1021/ja905732q

[b44] Chen G. . Synthesis of scaly Sn_3_O_4_/TiO_2_ nanobelt heterostructures for enhanced UV-visible light photocatalytic activity. Nanoscale 7, 3117–3125 (2015). 25611372 10.1039/c4nr05749j

[b45] Ahmed Y., Zahira Y. & Akhtar Parul. Degradation and mineralization of methylene blue using a heterogeneous photo-Fenton catalyst under visible and solar light irradiation. Catal. Sci. Tech. 6, 1222–1232 (2016).

[b46] Qiu Bocheng . Stöber-like method to synthesize ultradispersed Fe_3_O_4_ nanoparticles on graphene with excellent photo-Fenton reaction and high-performance lithium storage. Appl. Catal. B 183, 216–223 (2016).

[b47] Herrmann J. M. . Characterization and photocatalytic activity in aqueous medium of TiO_2_ and Ag-TiO_2_ coatings on quartz. Appl. Catal. B 13, 219–228 (1997).

[b48] Lin X., Huang T., Huang F., Wang W. & Shi J. Photocatalytic activity of a Bi-based oxychloride Bi_3_O_4_Cl. J. Phys. Chem. B 110, 24629–24634 (2006). 17134224 10.1021/jp065373m

[b49] Kim Y. I., Atherton S. J., Brigham E. S. & Mallouk T. E. Sensitized layered metal oxide semiconductor particles for photochemical hydrogen evolution from nonsacrificial electron donors. J. Phys. Chem. B 97, 11802–11810 (1993).

[b50] Leng W. H., Zhang Z., Zhang J. Q. & Cao C. N. Investigation of the kinetics of a TiO_2_ photoelectrocatalytic reaction involving charge transfer and recombination through surface states by electrochemical impedance spectroscopy. J. Phys. Chem. B 109, 15008–15023 (2005). 16852900 10.1021/jp051821z

[b51] Feng J., Hu X. & Yue P. L. Novel bentonite clay-based Fe-nanocomposite as a heterogeneous catalyst for photo-Fenton discoloration and mineralization of orange II. Environ. Sci. Technol. 38, 269–275 (2004). 14740746 10.1021/es034515c

[b52] Bacardit J., Stötzner J., Chamarro E. & Esplugas S. Effect of salinity on the photo-Fenton process. Ind. Eng. Chem. Res. 46, 7615–7619 (2007).

[b53] Zhai T. . Oxygen vacancies enhancing capacitive properties of MnO_2_ nanorods for wearable asymmetric supercapacitors. Nano Energy 8, 255–263 (2014).

[b54] Fu C., Mahadevegowda A. & Grant P. S. Production of hollow and porous Fe_2_O_3_ from industrial mill scale and its potential for large-scale electrochemical energy storage applications. J. Mater. Chem. A 4, 2597–2604 (2016).

[b55] Zhu L. . Core–shell MnO_2_@ Fe_2_O_3_ nanospindles as a positive electrode for aqueous supercapacitors. J. Mater. Chem. A 3, 22066–22072 (2015).

[b56] Arul N. S., Mangalaraj D., Ramachandran R., Grace A. N. & Han J. I. Fabrication of CeO_2_/Fe_2_O_3_ composite nanospindles for enhanced visible light driven photocatalysts and supercapacitor electrodes. J. Mater. Chem. A 3, 15248–15258 (2015).

[b57] Javed M. S. . High performance solid state flexible supercapacitor based on molybdenum sulfide hierarchical nanospheres. J. Power Sources 285, 63–69 (2015).

[b58] Ilanchezhiyan P., Kumar G. M. & Kang T. W. Electrochemical studies of spherically clustered MoS_2_ nanostructures for electrode applications. J. Alloy. Compd. 634, 104–108 (2015).

[b59] Wang L., Ma Y., Yang M. & Qi Y. Hierarchical hollow MoS_2_ nanospheres with enhanced electrochemical properties used as an electrode in supercapacitor. Electrochim. Acta 186, 391–396 (2015).

