# Supplementary material for: High Efficient Photo-Fenton Catalyst of α-Fe2O3/MoS2 Hierarchical Nanoheterostructures: Reutilization for Supercapacitors
Source: Sci Rep. 2016 Aug 16;6:31591. doi: 10.1038/srep31591 (PMC4985694; doi:10.1038/srep31591)
Supplement: Supplementary Information [file srep31591-s1.pdf]

Supplementary Information

High Efficient Photo-Fenton Catalyst of α-Fe_2O_3/MoS_2 Hierarchical Nanoheterostructures: Reutilization for Supercapacitors

Xijia Yang, Haiming Sun, Lishu Zhang, Lijun Zhao*, Jianshe Lian* and Qing Jiang

Key Lab of Automobile Materials, Ministry of Education, College of Materials Science and Engineering, Jilin University, Nanling Campus, Changchun, 130025, P.R. China.

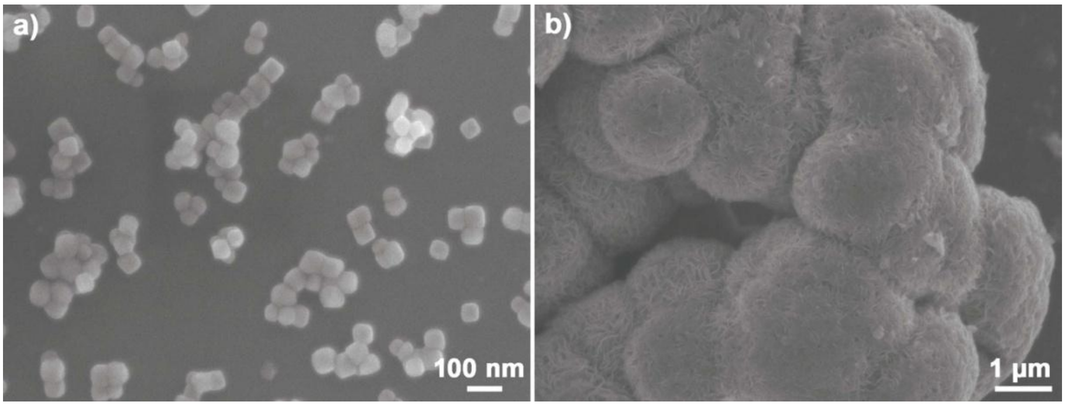

Figure S1. SEM images of a) pure Fe_2O_3 nanoparticles, b) pure MoS_2 nanosheets.

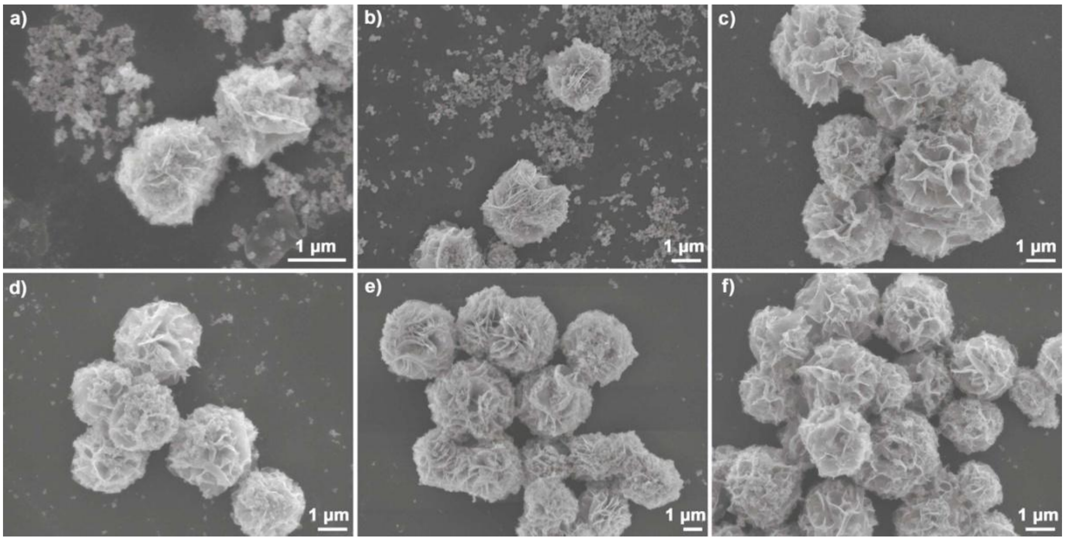

Figure S2. FE-SEM images of Fe_2O_3/MoS_2 heterostructures with different proportions: a) 0.6MF, b) 1.0MF, c) 1.4MF, d) 2.0MF, e) 3.0MF, f) 4.0MF.

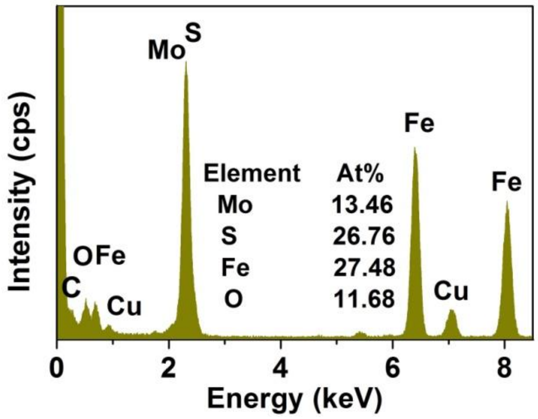

Figure S3. EDX patterns of Fe_2O_3/MoS_2 heterostructures. (The signals of Cu and C in the EDS spectrum originate from the carbon-coated copper grid.)

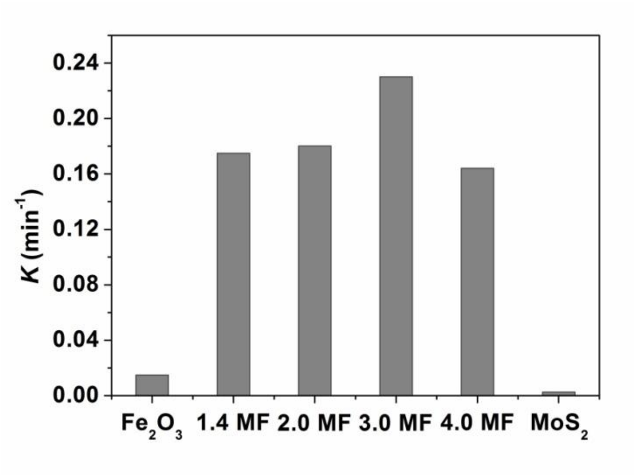

Figure S4. The rate constant k values for Fe_2O_3, 1.4MF, 2.0MF, 3.0MF, 4.0MF and MoS_2 under simulated solar light irradiation.

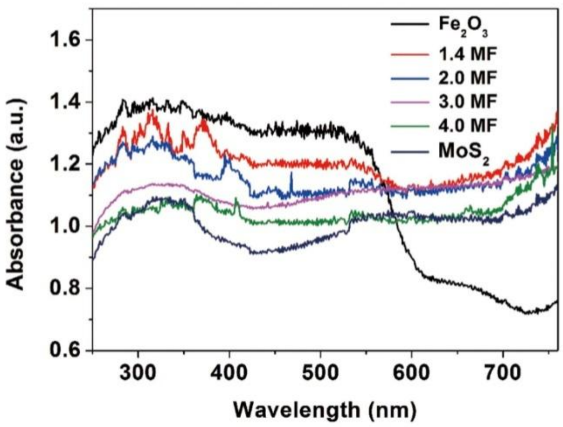

Figure S5.UV-Vis absorption spectra ofthe samples.

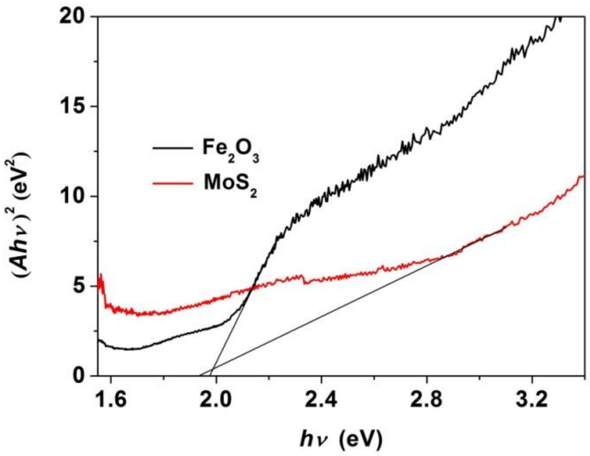

Figure S6. $(Ah\nu)^2$ vs $h\nu$ curves from absorption spectra to get band gap values for Fe_2O_3 nanoparticles and MoS_2 nanosheets.

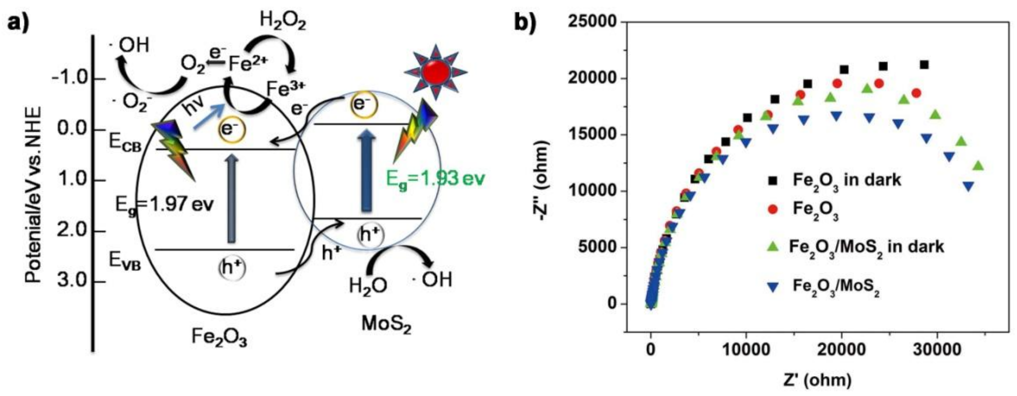

Figure S7. a) Schematic illustration of the charge separation for Fe_2O_3/MoS_2 heterostructures under simulated solar light; b) EIS Nyquist plots of the A: Fe_2O_3, B: Fe_2O_3 in dark, C: MoS_2, D: MoS_2 in dark, E: Fe_2O_3/MoS_2, and F: Fe_2O_3/MoS_2 in dark.

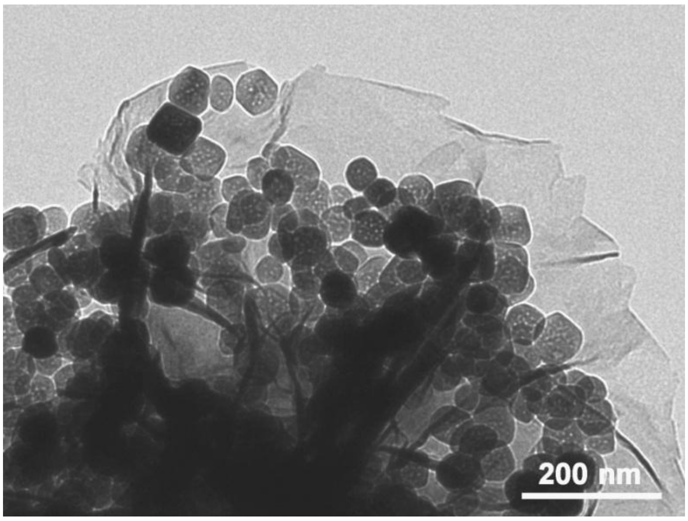

Figure S8. TEM image of the as prepared 3.0MF.

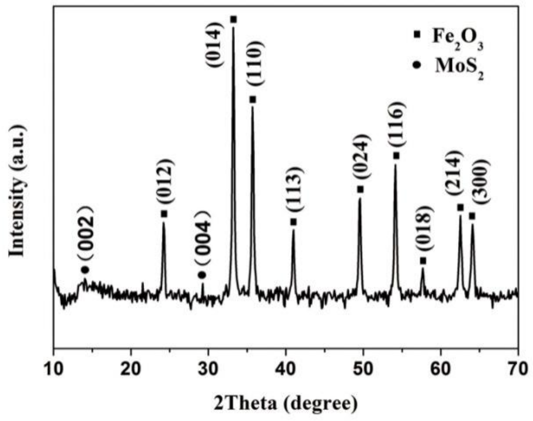

Figure S9. XRD patterns of the Fe_2O_3/MoS_2heterostructures (3.0MF) after 6 cycles of photocatalytic measurement.

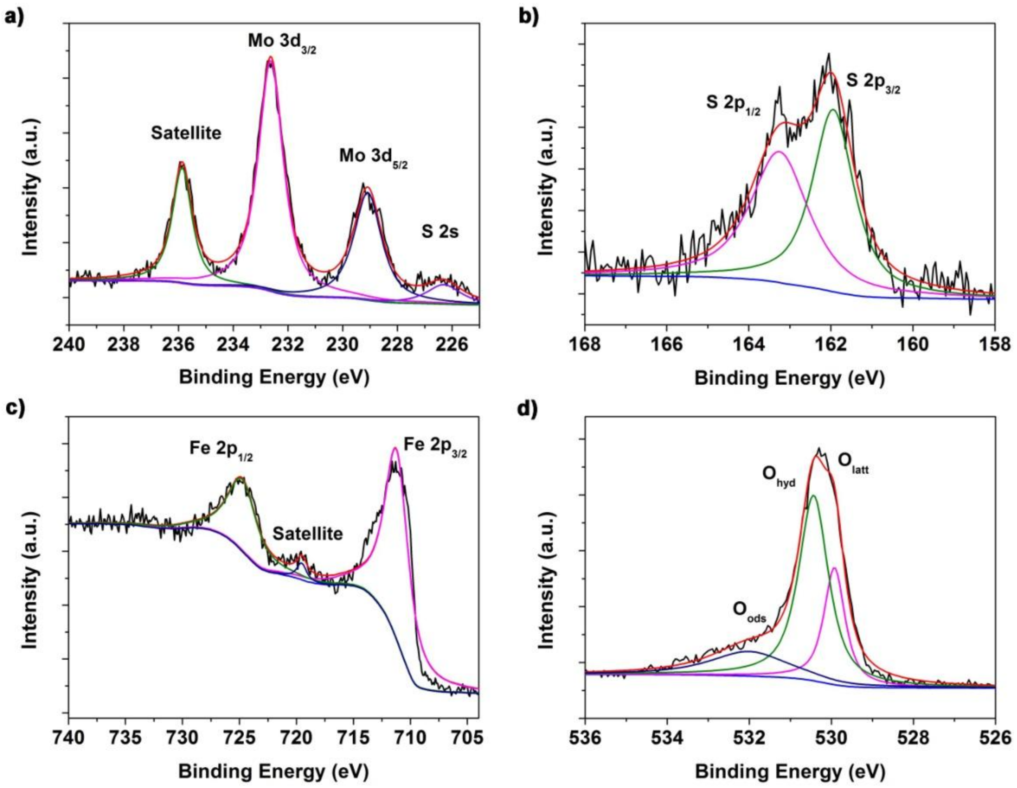

Figure S10. XPS spectra of the recycled 3.0MF: a) Mo 3d and S 2s peaks, b) S 2p peaks, c) Fe 2p peaks, and d) O 1s peaks.

Table S1. Comparison of photocatalytic activity of different materials for organic dyes degradation.

Photocatalysts	Light source	Photocatalyst	Organic dyes	%degradation	Literature
α-Fe_2O_3/MoS_2	Simulated solar light	10 mg/30 mL	Methyl orange (0.020 g L^{-1})<br>Congo red (0.050 g L^{-1})<br>Rhodamine B (0.020 g L^{-1})	99 (10 min)<br>96.7 (8 min)<br>99.7 (8 min)	Present work
α-Fe_2O_3/Graphene	Simulated solar light	30 mg	Rhodamine B (0.010 g L^{-1})	98 (20 min)	[1]
Ag_2O/TiO_2/V_2O_5	Simulated solar light	20 mg/30 mL	Rhodamine B (0.010 g L^{-1})	99.5 (60 min)	[2]
C/TiO_2	Simulated solar light	10 mg/30 mL	Methyl blue (0.020 g L^{-1})	Close to100 (30 min)	[3]
Sn_3O_4/TiO_2	Simulated solar light	30 mg/30 mL	Methyl orange (0.020 g L^{-1})	Close to100 (40 min)	[4]
hrGO/γ- Fe_2O_3	UV light	10 mg/100 mL	Methyl blue (0.10 g L^{-1})	Close to 100 (3h)	[5]
Fe–Ni/SiO_2	Simulated solar light	85 mg/100 mL	Methyl blue (0.020 g L^{-1})	Close to100 (60 min)	[6]
Fe_3O_4/RGO	Simulated solar light	Not mentioned	Methyl orange (0.010 g L^{-1})	98 (30 min)	[7]

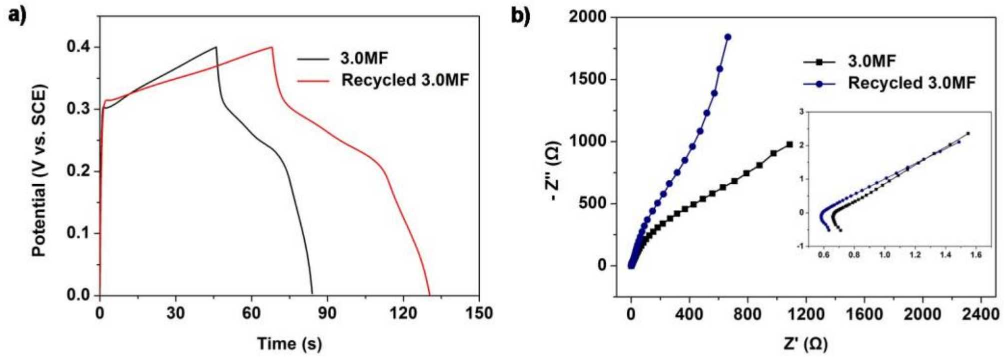

Figure S11. a) Galvanostatic charge-discharge curves at a current density of 1A g^{-1} for 3.0MF and recycled 3.0MF; b) EIS spectra of the 3.0MF and recycled 3.0MF.

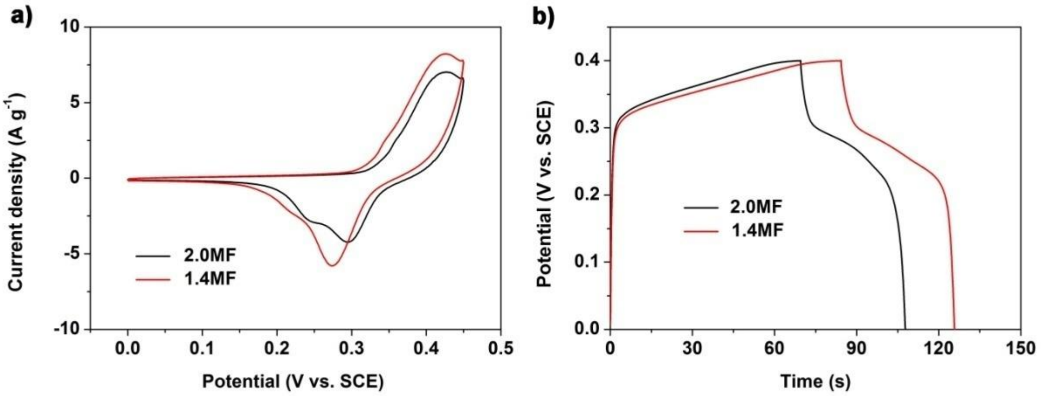

Figure S12. a) CV curves of the 2.0MF and1.4MF at a scan rates 20 mv s^{-1}; b) Galvanostatic charge-discharge curves at a current density of 1A g^{-1} for 2.0MF and 1.4MF.

References

1. Han, S. et al. One-step hydrothermal synthesis of 2D hexagonal nanoplates of α-Fe_2O_3/graphene composites with enhanced photocatalytic activity. *Adv. Funct. Mater.* **24**, 5719-5727 (2014).

2. Wang,Y. et al. $Ag_2O/TiO_2/V_2O_5$ one-dimensional nanoheterostructures for superior solar light photocatalytic activity. *Nanoscale* **6**, 6790-6797 (2014).

3 Wang, S. et al. Enhancing photocatalytic activity of disorder-engineered C/TiO_2 and TiO_2 nanoparticles. *J. Mater. Chem. A* **2**, 7439-7445 (2014).

4 Chen, G. et al. Synthesis of scaly Sn_3O_4/TiO_2 nanobelt heterostructures for enhanced UV-visible light photocatalytic activity. *Nanoscale* **7**, 3117-3125 (2015).

5. Yun, S., Lee, Y. C. & Park, H. S. Phase-controlled iron oxide nanobox deposited on hierarchically structured graphene networks for lithium ion storage and photocatalysis. Sci. rep. **6**, 1-9 (2016).

6. Ahmed, Y., Zahira Y. & and Parul Akhtar. Degradation and mineralization of methylene blue using a heterogeneous photo-Fenton catalyst under visible and solar light irradiation. Catal. Sci. Tech. **6**, 1222-1232 (2016).

7. Qiu, Bocheng, et al. Stöber-like method to synthesize ultradispersed Fe_3O_4 nanoparticles on graphene with excellent photo-Fenton reaction and high-performance lithium storage. Appl. Catal. B **183** 216-223 (2016).
